# Complete sequence of wild *Physalis philadelphica* chloroplast genome

**DOI:** 10.1080/23802359.2019.1673231

**Published:** 2019-10-01

**Authors:** Isaac Sandoval-Padilla, Jessica Pérez-Alquicira, María del Pilar Zamora-Tavares, Aarón Rodríguez, Moisés Cortés-Cruz, Gabriela Alcalá-Gómez, Ofelia Vargas-Ponce

**Affiliations:** a Maestría en Biosistemática y Manejo de Recursos Naturales y Agrícolas, Centro Universitario de Ciencias Biológicas y Agropecuarias, Universidad de Guadalajara, Zapopan, México;; b Cátedras CONACyT–Universidad de Guadalajara, Laboratorio Nacional de Identificación y Caracterización Vegetal (LaniVeg), Departamento de Botánica y Zoología, Centro Universitario de Ciencias Biológicas y Agropecuarias, Universidad de Guadalajara, Zapopan, México;; c Laboratorio Nacional de Identificación y Caracterización Vegetal (LaniVeg), Consejo Nacional de Ciencia y Tecnología (CONACyT), Universidad de Guadalajara, Zapopan, México;; d Instituto de Botánica, Depto. Botánica y Zoología, Centro Universitario de Ciencias Biológicas y Agropecuarias, Universidad de Guadalajara, Zapopan, México;; e Centro Nacional de Recursos Genéticos, Laboratorio de ADN y Genómicas, Instituto Nacional de Investigaciones Forestales, Agrícolas y Pecuarias, Tepatitlán de Morelos, México;; f Doctorado en Biosistemática, Ecología y Manejo de Recursos Naturales y Agrícolas, Centro Universitario de Ciencias Biológicas y Agropecuarias, Universidad de Guadalajara, Zapopan, México

**Keywords:** Annual crops, husk tomato, plastome, phylogeny, Solanaceae

## Abstract

*Physalis philadelphica* Lam. has horticultural importance because of its edible fruits. Cultivated and wild populations grow in Mexico. In this study, the complete plastome nucleotide sequence of wild plants was generated using the IonTorrent PGM sequencing technology. The plastome size was 156,804 bp and displayed the typical circular quadripartite structure, consisting of a pair of inverted repeat regions (25,595 bp) separated by a large single copy region (87,131 bp) and a small single copy region (18,483 bp). The chloroplast genome included 80 protein–coding genes, four rRNAs, and 31 tRNAs. The phylogenetic analysis based on 19 Solanaceae chloroplast genomes recovered a clade with all *Physalis* species. This work revealed the importance of the plastome sequence to solve infrageneric phylogenetic relationships.

The genus *Physalis* L. (Solanaceae) groups 90 species and Mexico is the centre of diversification (Zamora-Tavares et al. 2015). Sixty-five species grow in Mexico, but *Physalis philadelphica* Lam. has commercial value. Its fruits are part of the staple diet of the Mexicans (Zamora-Tavares et al. 2015). Wild populations of *Physalis philadelphica* grow nationwide, across different environments (Martínez et al. 2017). It is cultivated in different regions of the country throughout the year, and in countries of the Americas, Europe, and Asia is present as an experimental crop (Zamora-Tavares et al. 2015).

Domestication reduces genetic variation but wild populations restore it (Casas et al. 2007). Across Angiosperms, chloroplast DNA has a conserved genomic structure with a high mutation rate in some regions (Ravi et al. 2008). As a result, plastome sequences are useful to understand relationships between domesticates and wild (Daniell et al. 2016). Plastome variation between cultivated and wild ancestors, as well as their comparison with other wild relatives, have been documented in plants of economic importance such as *Oryza spp.* (*O. barthii* A.Chev, *O. glaberrima* Steud., *O. nivara* S.D.Sharma & Shastry, *O. rufipogon* Griff., *O. sativa* L., Tong et al. 2016) and *Helianthus annuus* L. (Makarenko et al. 2016). It is our interest to make these comparisons in *P. philadelphica* to elucidate if the species has undergone different evolutionary patterns. In the present study, we sequenced and assembled the complete plastome of wild plants of *P. philadelphica*. Until now, GenBank has three available plastomes from *Physalis* (gene bank accession numbers *P. angulata* L. NC039457, *P. peruviana* L. MH019242, and *P. pruinosa* L. NC039458).

Fresh leaves from wild plants of *Physalis philadelphica* were harvested in San Antonio de los Vázquez, Ixtlahuacán del Río, Jalisco, México (20°49′29.14″N, 103°7′39.55″O). cpDNA was isolated based on Shi et al. (2012) and was stored at the Laboratorio Nacional de Identificación y Caracterización Vegetal (LaniVeg) at the Universidad de Guadalajara (Voucher: 021118ISP). A total of 1,685,803 single-end reads were generated using the IonTorrent PGM technology (Thermo Fisher Scientific, Carlsbad, CA) and these reads were *de novo* assembled into contigs using SPAdes 3.12.0 (Bankevich et al. 2012). The assembled genomic sequences were filtered with Bowtie2 2.3.5 (Langmead and Salzberg 2012) followed by the annotation using DOGMA (Wyman et al. 2004). tRNAs were predicted with tRNAscan SE 2.0.3 (Chan and Lowe 2019). The circular representation of the *P. philadelphica* plastome (GenBank MN192191) was obtained with OGDraw 1.3.1 (Greiner et al. 2019).

The chloroplast genome size of *Physalis philadelphica* was 156,804 bp. The genome displayed the typical circular quadripartite structure. It consisted of a pair of inverted repeated (IR) regions (25,595 bp) separated by two single copy regions, one large (LSC, 87,131 bp) and one small (SSC, 18,483 bp). Plastome nucleotide composition was 30.87% adenine, 19.06% cytosine, 18.45% guanine, and 31.66% thymine. General guanine-cytosine content was 37.5%, but the IR had 43.06% each, and the LSC and SSC regions showed 35.56 and 31.35%, respectively. The plastome included 115 genes, 80 protein-coding, four rRNAs, and 31 tRNAs. Twenty-two duplicate genes were present in the IR regions: four rRNAs, seven tRNAs, nine protein-coding, and two pseudogenes. Additionally, there were 15 genes harbouring introns, 13 with two and two with one.

Within Solanaceae, the phylogenetic position of *Physalis philadelphica* was estimated. The phylogenetic tree was constructed based on 17 Solanaceae chloroplast genomes available on GenBank, using *Ipomoea batatas* (L.) Lam. (Convolvulaceae) as outgroup. Maximum Likelihood (ML) analysis was performed using RA × ML (Stamatakis, 2014) and 1,000 bootstraps iterations. The phylogenetic estimation was performed with the evolutionary model GTR + I + G, according to the estimate of jModelTest 2.1.10 (Darriba et al. 2012). At the tribe level, the cladogram recovered the same relationships of Solanaceae as did Särkinen et al. (2013). Subtribes Iochrominae and Physalidinae had a sister group relationship (Deanna et al. 2019). The phylogenetic hypothesis supported *Physalis* as a monophyletic group (Figure 1). Even though, the statistical support was moderated, this work revealed the usefulness of the plastome sequence to solve infrageneric phylogenetic relationships.

**Figure 1.**
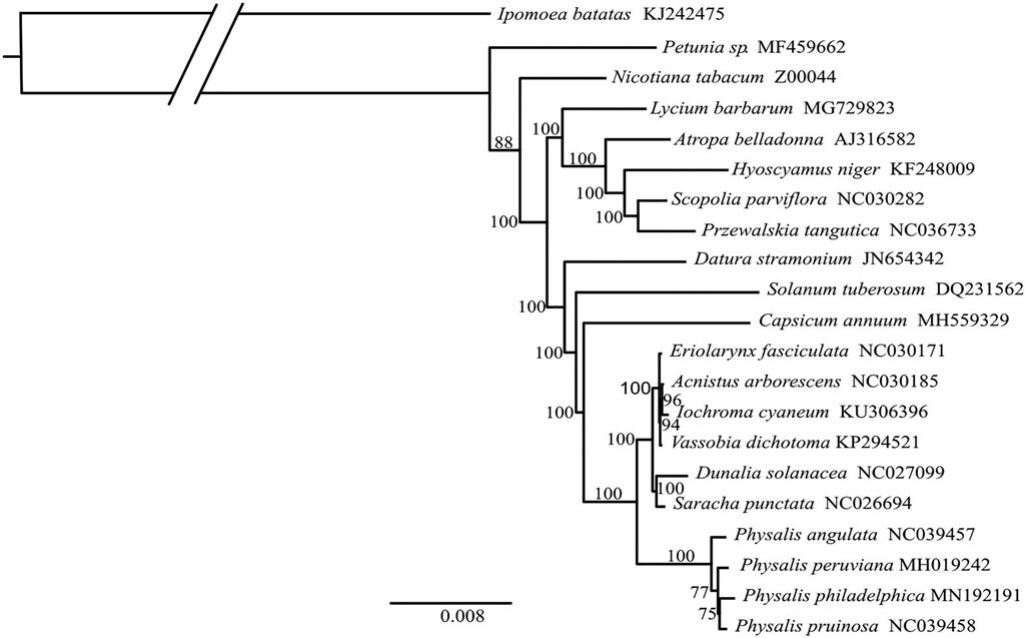
The maximum-likelihood (ML) tree of 20 Solanaceae species including *Physalis philadelphica*. Bootstrap value based on 1000 replicates are shown in the nodes. GenBank accession numbers are shown after the species name.
